# Construction and evaluation of a whole genome microarray of *Chlamydomonas reinhardtii*

**DOI:** 10.1186/1471-2164-12-579

**Published:** 2011-11-25

**Authors:** Jörg Toepel, Stefan P Albaum, Samuel Arvidsson, Alexander Goesmann, Marco la Russa, Kristin Rogge, Olaf Kruse

**Affiliations:** 1 Algae Biotechnology & Bioenergy, Dept. of Biology, Center for Biotechnology (CeBiTec), Bielefeld University, 33615 Bielefeld, Germany; 2 Computational Genomics, Center for Biotechnology (CeBiTec), Bielefeld University, 33615 Bielefeld, Germany; 3 AG Bioinformatics, GoFORSYS, Max Planck Institute of Molecular Plant Physiology, 14476 Potsdam-Golm, Germany

## Abstract

**Background:**

*Chlamydomonas reinhardtii *is widely accepted as a model organism regarding photosynthesis, circadian rhythm, cell mobility, phototaxis, and biotechnology. The complete annotation of the genome allows transcriptomic studies, however a new microarray platform was needed. Based on the completed annotation of *Chlamydomonas reinhardtii *a new microarray on an Agilent platform was designed using an extended JGI 3.1 genome data set which included 15000 transcript models.

**Results:**

In total 44000 probes were determined (3 independent probes per transcript model) covering 93% of the transcriptome. Alignment studies with the recently published AUGUSTUS 10.2 annotation confirmed 11000 transcript models resulting in a very good coverage of 70% of the transcriptome (17000). Following the estimation of 10000 predicted genes in *Chlamydomonas reinhardtii *our new microarray, nevertheless, covers the expected genome by 90-95%.

**Conclusions:**

To demonstrate the capabilities of the new microarray, we analyzed transcript levels for cultures grown under nitrogen as well as sulfate limitation, and compared the results with recently published microarray and RNA-seq data. We could thereby confirm previous results derived from data on nutrient-starvation induced gene expression of a group of genes related to protein transport and adaptation of the metabolism as well as genes related to efficient light harvesting, light energy distribution and photosynthetic electron transport.

## Background

*Chlamydomonas reinhardtii *is widely accepted as a model organism regarding photosynthesis, circadian rhythm and biotechnology for several decades. With the first design of a *C. reinhardtii *microarray [1], transcriptomic analyses in this organism could be conducted. This first generation microarray contained 10000 transcript models with 8667 of them being associated with current transcript models covering about 87% of the predicted genome [2] with nearly 10000 genes. However, rapid progress in genome annotation [3] resulted in improved transcriptome data [4] which clearly demanded the design of a new microarray platform for advanced and general transcriptome analyses. Microarrays are relatively cheap and reliable systems to analyze transcript levels on a routine basis and they are perfectly complementary to the recently established RNA-seq platforms [5]. The advantages of RNA-seq are manifold, e.g. the higher gene coverage and the increased sensitivity for differential gene expression [5-10]. The characterization of new gene models and splicing variations are easier to predict, as well as the detection and characterization of mutation sites [11]. However, the results of RNA-seq are still critical to examine and high reproducibility is often difficult to achieve. As a typical consequence, an overestimation of high abundant genes and length dependent amplification has been reported using RNA-seq [12-14]. These internal biases are still under discussion and data analysis and data normalization clearly need to be improved. Additional advantages of microarrays compared to RNA-seq are still the significant lower costs (between 10-100 times) and the good coverage of exon based transcript levels, with around 90% [13], where extremely deep sequencing would be necessary in order to achieve the same transcript coverage with RNA-seq. Furthermore, microarray experiments are less time consuming, allow the run of multiple replicates and established analysis platforms for routine transcript level analyses are available. However, the current microarray platform [1], with 10000 features, covers just 87% of the predicted genome and many newly annotated genes are missing [2]. Based on estimations using the *Chlamydomonas *genome, up to 17000 transcript models are expected to be present in this green algae [4,15].

Generally, *C. reinhardtii *adaption to varying stress conditions can be best evaluated by using -omics approaches. Transcriptome studies were performed by different applications, e.g. microarray or RNA-seq, during nutrient starvation [16-18], anaerobiosis [19], hydrogen production [20], oxidative [21] or light stress [22]. The induction of genes responding to nutrient starvation, e.g. sulfur and nitrogen starvation is well documented and available data sets are used within this work to test the reliability of our newly designed microarray. A recent study investigating the effect of sulfur starvation [16] included a comparison of RNA-seq data with those acquired from microarray studies. The data comparison showed a good accordance between both methods. Adaptation to sulfur stress starts with an induction of genes responsible for nutrient transport accompanied by the repression of gene expression related to photosynthetic processes. In a later step, acclimatization/modulation processes include changes in the amino acid composition [23] of certain target proteins and the synthesis of starch [2]. Furthermore, lipid metabolism was shown to be affected under sulfur starvation caused by the physiological shift to anaerobic conditions [23,24]. In *C. reinhardtii *it is of particular interest that under sulfur depletion, plastidial hydrogenase activity and consequently hydrogen production is strongly induced [25,26]. Therefore, we also used existing transcriptome data sets of experiments to confirm gene expression pattern under sulfur starvation. So far, a number of studies employing high-throughput technologies including transcriptomics, proteomics and metabolomics have been carried out to describe the process of hydrogen production in *C. reinhardtii *[20,24,27].

Another well documented stress condition is the growth of *C. reinhardtii *under nitrogen starvation [28-30]. Recent analyses of gene expression variation under nitrogen starvation by RNA-seq [17] precisely describe adaptation processes of photosynthesis as well as of anabolic metabolism mechanisms (lipid and amino acid production). The high sensitivity of RNA-seq was demonstrated by monitoring differences in expression rates of low abundant genes coding for transcripts involved in regulatory processes. In detail, it could be concluded that nitrogen starvation results in a decreased photosynthetic gene expression and activity, increased lipid accumulation and induction of gametogenesis.

In this present study, we performed microarray experiments with our newly developed *C. reinhardtii *full genome microarray to prove its suitability for differential transcript analyses and for comparing time-course global expression profiles of *C. reinhardtii *under starvation conditions. Additionally, we tested the sensitivity of the array for identifying knockout mutations.

## Results

### Design

**Figure 1:**
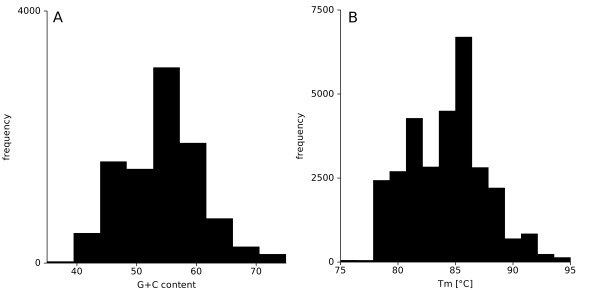
Chemical properties of the designed transcript probes for a *Chlamydomonas reinhardtii *4*44 k *Agilent *microarray: (A) Histogram of the G+C content and (B) Histogram of the melting temperature (Tm).

Microarray design was based on data sets provided by the JGI 3.1 genome annotation of *C. reinhardtii*. This new *Chlamydomonas reinhardtii *microarray platform is now available under the Agilent^© ^access number 024664. The microarray design is, however, not fixed: a crucial advantage is that newly annotated genes can easily be added to this array. The adapted new transcriptome consists of 15000 annotated nucleus-encoded gene models. We designed 60 mer oligonucleotides using two software tools: ARRAY-EXPRESS^© ^and PROMIDE^©^. The detailed origin for each probe and probe sequence is summarized in additional file 1, Table S1. Both software tools were used to design temperature and position optimized probes (5'-3' bias). As a result, we determined sequence optimized probes for 14557 gene models, which represent 93% coverage of the transcriptome based on the JGI3.1 genome annotation. We were unable to determine specific probes for the remaining transcript models. In Figure 1, the chemical properties of the designed probes are described. The G/C content of the probes varied between 40 and 60% (Figure 1A) and melting temperature distribution for all probes was between 80 and 90°C, (Figure 1B).

Additionally, we used 8760 approved sequences from the first generation *C. reinhardtii *microarray, adapted to the Agilent^© ^platform [3] and added the probes as a third replicate to our microarray platform. However, according to our BLAST analysis (reference: AUGUSTUS 10.2) are just 7200 probes specific for one transcript model and we used just proved probes for our new microarray platform.

Finally, we compared the new determined probe sequences with the new published annotation (AUGUSTUS 10.2) and could confirm *in silico *by BLAST analysis http://blast.ncbi.nlm.nih.gov/ the hybridization specificity for 70% of the 14557 transcript specific sequences. The remaining probes showed potential cross hybridization properties (7%, with more than 3 mismatches in the sequence) or could not be aligned to current transcripts (23%). It should be noted that the genome annotation of the *C. reinhardtii *genome is not yet finished and the final number of transcript models is still under discussion. Detailed information about the specificity and potential cross hybridization targets is provided in additional file 2, Table S2.

### Testing

**Figure 2:**
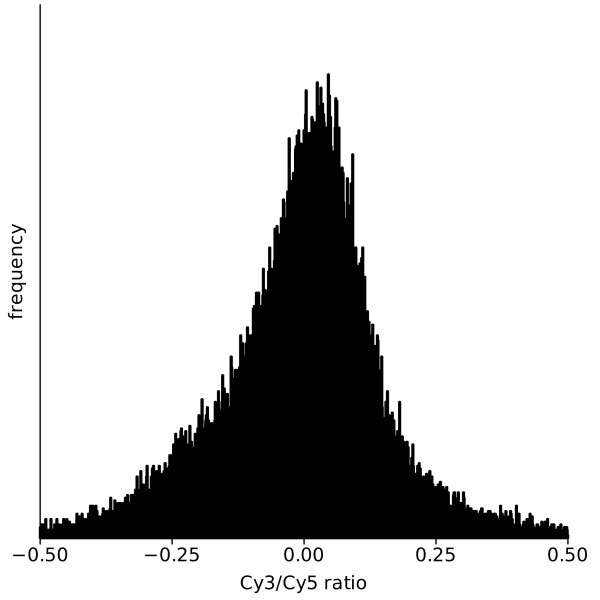
**Testing of the Cy-3 and Cy-5 labeling pre-correction with the feature extraction software (10.7.3.1.), using identical RNA samples of *Chlamydomonas reinhardtii***.

We used identical RNA samples from cells grown for 24 h under sulfur starvation to check Cy-3 and Cy-5 labeling. Successful pre-correction was achieved with the feature extraction software (10.7.3.1). As a result we could demonstrate that labeling is nearly identical with both dyes. The Cy3/Cy5 log 2 ratio showed a good distribution around 0 and therefore an additional dye-specific correction is not necessary (Figure 2). To ensure that any remaining differences between the two labeling dyes are equalized, all computed log2 ratios were normalized. Following the recommendations of [31], a normalization method based on robust local regression (lowess) was utilized for this purpose.

**Figure 3:**
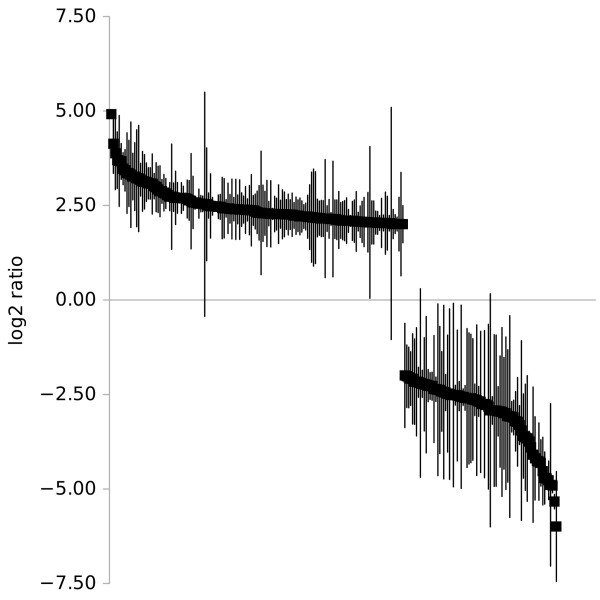
**Average values and standard deviations of Cy3/Cy5 log2 ratios from replication experiment (6 times) with *Chlamydomonas reinhardtii *RNA**.

Normalization and analysis were carried out with the in-house developed EMMA2 software [32]. To test the reproducibility of the data, a 6 fold replication with labeled RNA samples taken from starvation experiments was performed. The high similarity of the data sets with a log2 ratio variation between 20 and 35% for all differential expressed genes derived from three biological and three technical replicas demonstrated the robustness of the system (Figure 3). The internal *C. reinhardtii *specific control probes showed a variation smaller than 10% (data not shown) in all experiments and could be therefore considered as a reliable control parameter for further experiments.

### Starvation experiments

We performed sulfur and nitrogen starvation experiments with *C. reinhardtii *WT *cc3491 *to analyze the expression of genes responding to nutrient starvation. As a result 25000 probes showed a significant fluorescence signal against the background, and were therefore used for data analysis. With three independent probes per transcript, around 8000 transcript models could be analyzed in the experiments. It should be noted that results derived from the newly designed probes showed a good concordance. However data derived from the probes of the first generation array showed a lower log2 ratio in differentially expressed genes and blast analysis showed a high cross hybridization potential for many probes. We therefore decided not to include these data sets into the analysis. The data analysis resulted in the identification of a relatively small number of genes responding to the respective nutrient limitation conditions. We performed a cluster analysis using the software GENESIS^© ^(hierarchical cluster analysis using Ward and Euclidean distances) for differentially regulated genes, to detect time-dependent gene expression in response to nutrient stress, and could conclude that the majority of the genes showed a fast response to the nutrient stress with constant expression level over the whole time-course. The late-responding genes mainly belong to transcripts related to secondary effects like phosphorus stress or CO_2 _limitation.

### Nitrogen starvation

The response of gene expression during nitrogen starvation was in general higher and faster than during sulfur starvation conditions. Around 200 genes showed twofold increased expression levels whereas approximately 300 genes were detected with a twofold reduced expression (additional file 3, Table S3). Comparison with RNA-seq transcriptome data derived from nitrogen starvation experiments [17] revealed a very similar picture with an identical differential gene expression pattern for 60% of the transcripts. In [17] transcripts were analyzed using Illumina Solexa^© ^combined with the 454^© ^ultra-fast-sequencing which resulted in the identification of several more transcripts. Differences in the two datasets are most likely a result of differences in time length of starvation (72 h instead of 48 h) and of differences in the intensity of illumination (200 μmol m^-2 ^s^-1 ^instead of 80 μmol m^-2 ^s^-1^). Another reason could be the possible bias by overestimation of large transcripts and differences in the range of detection. Within our experiments we were able to confirm the up-regulation of components of the nitrogen transport systems, such as ammonia-, nitrate- and nitrite-transporters (see Table 1). From 7 annotated ammonium transporters (AMT), only *AMT4 *and *AMT1 *were up-regulated. This result was achieved with all three probes per transcript with a minimal log2 ratio of 4 and shows high similarities to earlier data provided by [17], however here some of the AMT transcripts were most likely miss-annotated. The nitrate transporter with the highest expression level was encoded by the locus Cre09.g410850.t1.1 As expected, the major nitrate transporter did not response to nutrient stress, since ammonia was provided as the nitrogen source in the growth medium. Furthermore, the up-regulation of one of the four annotated glutamine synthetases (*GLN3*, Cre12.g530600.t1.1) was confirmed, no up-regulation of *GLN1 *(Cre02.g113200.t1.1); *GLN2 *(Cre12.g530650.t1.1) and *GLN4 *(Cre03.g207250.t1.1) was detectable, similar results were obtained from the RNA seq data. Additionally, increased gene expression for one nitrite reductase (Cre09.g410750.t1.1) and one nitrate reductase (Cre09.g410950.t1.1) could be confirmed within our experiments. Interestingly, although under these conditions the majority of the photosynthetic gene transcripts were down-regulated, several genes related to photosynthesis showed an up-regulation in transcription rates. In detail, some genes responsible for PS light harvesting and energy distribution like *LHCA1*, *LHCSR3 *and several photosystem II subunits like *PSBX *and *PSBS1 *were up-regulated during nitrogen starvation. However, it is noteworthy and somewhat surprisingly that we detected at the same time down-regulation of *LHCSR1 *under nitrogen limitation, a result which is in good accordance to RNA-seq data. It has been reported that *LHSCR *genes are up-regulated under stress conditions and responsible for de-excitation of chlorophyll molecules in PS II [33,34]. Furthermore in good agreement with [17], none of the ribosomal related genes were up-regulated. We could further confirm the *NIT2 *induction (transcription factor regulating nitrogen metabolism) and the repression of *NAB1*, a nuclear encoded mRNA binding factor, which specifically binds and sequesters *LHCII *mRNA and prevents their translation [35-37]. Many carbonic anhydrases showed a down-regulation; however the mitochondrial carbonic anhydrase (Cre05.g248450.t1.1) was up-regulated. Regarding the TCA-cycle the transcript level of the citrate synthase (Cre12.g514750.t1.1) increased during nitrogen starvation, in contrast to the isocitrate lyase 1 (*ICL1*) which was not affected in our experiments. Additionally, our data indicated an up-regulation of several genes induced by phosphate starvation, like *PSR1 *(phosphorus starvation response 1 protein, transcriptional regulator) as earlier described [18]. PSR1 however, did not show an increased level of expression within the RNA-seq data. Again, the reason for the differences could be due to the longer starvation period leading to secondary effects.

**Table 1:** Comparison between RNA-seq [17] (48 h) and microarray data for *Chlamydomonas reinhardtii *cultures incubated under nitrogen starvation conditions For microarray data average values for important genes during 96 h starvation are displayed (24 h, 48 h, 72 h, 96 h).

Reporter Identifier	Reporter Name	Reporter Description	Log2 ratio microarray	Log2 ratio RNA seq
	**nitrogen related genes**			
182971	NSG13	"nitrogen-starved gametogenesis 13"...	5.0	3.6
184661	NIT1	"nitrate reductase"...	4.6	7.9
192085	NII1	"Nitrite reductase"...	4.4	7.7
205647	NIT2	"transcription factor regulating nitrogen metabolism"...	1.5	2.6
188890		"NADH nitrate reductase"...	3.0	5.2
156131	AMT4	"Ammonium transporter"...	5.1	7.2
158745	AMT1	"Ammonium transporter"...	3.1	5.5
133971	GLN1	Glutamine synthetase	0.9	1
129468	GLN2	Glutamine synthetase	0.1	-0.3
136895	GLN3	Glutamine synthetase	6.5	6.6
147483	GLN4	Glutamine synthetase	2.3	1
				
	**photosynthetic related genes**			
184724	LHCSR1	"stress-related chlorophyll a/b binding protein 1"...	-2.4	-1.2
184731	LHCSR2	"Stress-related chlorophyll a/b binding protein 2"...	3.7	2.6
184730	LHCSR3	"Stress-related chlorophyll a/b binding protein 3"...	3.8	1.7
				
196341	PSBS1	"chloroplast Photosystem II-associated 22 kDa protein"...	2.0	6.9
171516	PSBS2	"chloroplast Photosystem II-associated 22 kDa protein"...	-0.3	
196341	PSBX	"4.1 kDa photosystem II subunit"...	-2.1	6.9
205940	PSAL	"Photosystem I reaction center subunit XI"...	-2.4	0.0
192478	PSAK	"photosystem I reaction center subunit psaK"...	-2.3	-3.4
				
184810	LHCB4	"chlorophyll a-b binding protein of photosystem II"...	-2.4	-2.3
187025	LHCA8	"light-harvesting protein of photosystem I"...	-2.2	-4.6
192961	LHCA7	"light-harvesting protein of photosystem I"...	-2.5	-5.0
186299	LHCA6	"light-harvesting protein of photosystem I"...	-2.7	-5.4
153678	LHCA4	"light-harvesting protein of photosystem I"...	-2.6	-4.5
206001	LHCA1	"light-harvesting protein of photosystem I"...	-3.2	
184397	LHCBM3	"Light-harvesting complex II chlorophyll a-b binding protein M3"...	-2.0	-3.7
184479	LHCBM9	"chlorophyll a-b binding protein of LHCII"...	-0.2	-1.5
126810	NAB1	"nucleic acid binding protein"...	-1.6	-4.1
				
	**other genes**			
185841	RDP3	"Rhodanese domain phosphatase"...	2.4	-3.4
196438	PTB5	"sodium/phosphate symporter"...	5.3	0.6
196465	PTB5	"sodium/phosphate symporter"...	4.3	1.7
183357	PTB3	"sodium/phosphate symporter"...	3.8	-0.6
96789	GPD4	"Glycerol-3-Phosphate Dehydrogenase/Dihydroxyacetone-3-Phosphate Reductase"...	2.8	1.4
146945	GPD1	"Glycerol-3-Phosphate Dehydrogenase/Dihydroxyacetone-3-phosphate Reductase"...	3.6	4.1
182461	GLD2	"glucose-6-phosphate dehydrogenase"...	2.3	4.1
191987	ARG1	"N-acetyl-gamma-glutamyl-phosphate reductase"...	2.8	0.8
R:191668/1	ICL1	"isocitrate lyase"	0.1	-5.8

### Sulfur stress

**Figure 4:**
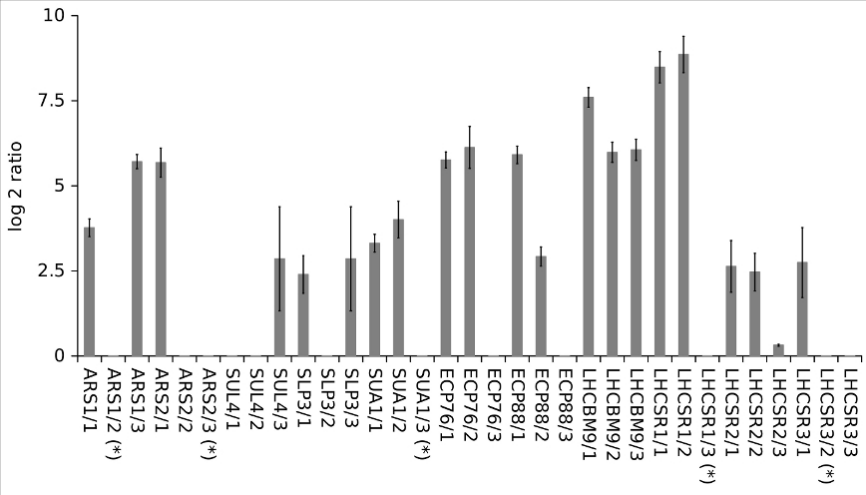
**Expression level for known sulfur induced genes in *Chlamydomonas reinhardtii*, displayed are the average values for 4 time points (24 h, 48 h, 72 h, 96 h) for the independent probes which showed a significant increased Log2 ratio for all time points**. Probes marked with a star are not specific for the transcript model.

Under sulfur starvation 813 genes showed a differential gene expression. 300 genes were down-regulated by at least 2-fold whereas around 100 genes were at least 2-fold up-regulated. Comparison between first generation and new microarray data derived from sulfur starvation experiments showed high similarities for many genes regarding changes in their differential gene expression pattern (for details see additional file 4, Table S4). We confirmed the up-regulation for several sulfate transport systems and induction of several stress response systems (Table 2). As shown in Figure 4 we were able to confirm the increased gene expression of two major aryl sulfatases (*ARS1 *and *ARS2*). Probe specificity for the other ARS enzymes could be confirmed but no increased gene expression was detectable. It should be noted that for ARS6, no probes were designed, since this gene has not yet been annotated at the time of the experiment. The extracellular proteins *ECP88*, *ECP76*, *SLP3 *(Sulfate binding protein), *SUA *(Chloroplast sulfate transporter) *STL1 *(sodium/sulfate co-transporter) and *SIR1 *(ferredoxin sulfite reductase 1) also showed an up-regulation for all determined and tested probes. Similar to the experiments with nitrogen starvation, most photosynthetic related genes were down-regulated however for several proteins involved in light harvesting and light quenching (*LHSBM9, LHCSR1, LHSCR3.1 *and *LHSCR3.2*) we could confirm an increase in transcript levels during all 4 time points.

**Table 2:** Comparison between RNA seq [16] (24 h) and microarray data for *Chlamydomonas reinhardtii *cultures incubated under sulfur starvation conditions For microarray data average values for the genes during 96 h starvation are displayed (24 h, 48 h, 72 h, 96 h).

Reporter Identifier	Reporter Name	Reporter Description	Log 2 ratio microarray	Log 2 ratio RNA seq
	**sulfur related genes**			
59800	SUOX1	"sulfite oxidase"...	3.5	2.0
205505	SLT3	"sodium/sulfate co-transporter"...	-4.5	-1.8
205506	SLT2	"sodium/sulfate co-transporter"...	2.1	-1.7
205507	SLT1	"sodium/sulfate co-transporter"...	3.1	6.9
206159	SIR1	"ferredoxin-sulfite reductase"...	1.7	2.0
55757	ARS2	"periplasmic arylsulfatase"...	5.4	8.2
205501	ARS1	"periplasmic arylsulfatase"...	4.3	6.3
133924	ATS2	"ATP-sulfurylase"...	4.0	2.8
175651	OASTL1 changed to ASL1		-1.2	-0.3
				
137329	ECP88	"88 kDa extracellular polypeptide"...	5.6	13.4
130684	ECP76	"76 kDa extracellular polypeptide"...	5.5	10.7
				
	**photosynthetic genes**			
182015	PSBX	"4.1 kDa photosystem II subunit"...	-3.1	-0.9
193847	PSAO	"Photosystem I subunit O"...	-3.5	-1.4
182959	PSAH	"Subunit H of photosystem I"...	-3.1	-1.4
187195	PRPL29	"Putative chloroplast ribosomal protein L29	-7.0	-2.3
				
184730	LHCSR3	"Stress-related chlorophyll a/b binding protein 3"...	3.9	2.3
184731	LHCSR2	"Stress-related chlorophyll a/b binding protein 2"...	2.4	2.1
184724	LHCSR1	"stress-related chlorophyll a/b binding protein 1"...	5.5	1.8
184479	LHCBM9	"chlorophyll a-b binding protein of LHCII"...	5.8	10.0
205757	LHCBM8	"chlorophyll a-b binding protein of LHCII"...	-2.8	-0.5
184071	LHCBM7	"chlorophyll a-b binding protein of LHCII"...	-2.6	-0.9
191690	LHCBM4	"chloropyll a-b binding protein of LHCII"...	-2.9	-0.3
184397	LHCB5	"minor chlorophyll a-b binding protein of photosystem II"	-3.2	-2.0
187025	LHCA8	"light-harvesting protein of photosystem I"...	-2.9	-1.3
183363	LHCA7	"light-harvesting protein of photosystem I"...	-3.5	-1.1
186299	LHCA5	"light-harvesting protein of photosystem I"...	-3.5	-1.4
184471	LHCA1	"light-harvesting protein of photosystem I"...	-3.1	-1.3
				
	**other genes**			
189430	CCP1	"low-CO2-inducible chloroplast envelope protein"...	-2.5	-0.7
194325	LCI33	"low-CO2-inducible protein"...	-2.3	1.7
55019	LCI1	"low-CO2-inducible membrane protein"...	-2.1	-2.9
				
97127	EFG1	"chloroplast elongation factor G"...	-2.9	-0.6
135322	CSP41b	"chloroplast stem-loop-binding protein"...	-2.5	0.4
205573	CSP41a	"chloroplast stem-loop-binding protein"...	-2.2	0.0
141844	CDJ4	"chloroplast DnaJ-like protein"...	2.6	0.8
				
134235	ATPC	"chloroplast ATP synthase gamma chain"...	-2.4	-0.4
81427	81427/1	"Chloroplast SRP54 Subunit of Signal Recognition Particle"...	2.2	-1.7
174103	IPY3	"soluble inorganic pyrophosphatase"...	-2.3	-2.8
148916	ELI3	"Early light-inducible protein"...	2.0	2.9

Overall, we found the majority of the genes (60%) previously described to be induced or repressed by sulfate starvation with the same expression pattern [16]. Variations can be explained by the different time scale of sample harvesting and different growth conditions which could also lead to secondary effects like phosphate limitation. Differences in the log2 ratios between RNA-seq data and microarray data are most likely a result of the higher dynamic range of the RNA-seq data, since saturation effects, which can occur for highly expressed genes on microarrays, are not expected.

Combing the array data sets derived from the two starvation experiments we identified several genes, which showed an increased or reduced transcript level under both nutrient stress conditions (additional file 5, Table S5). Most of these genes are either of unknown function or are reported to be involved in transport or metabolism.

### *LHCSR3 *knock out detection

**Figure 5:**
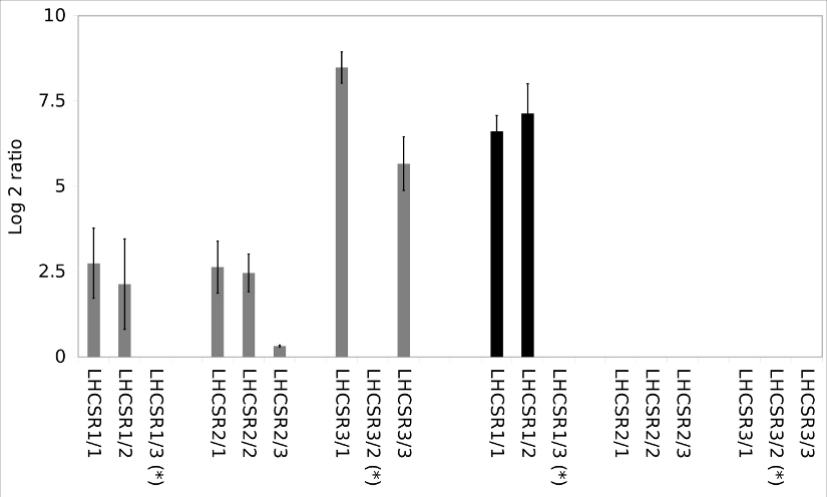
**Characterization of the expression level of *lhcsr *transcripts during sulfur starvation *Chlamydomonas reinhardtii *in *4A*+ (grey) and *npq4 *(black)**. Displayed are average values for the transcripts during 96 h starvation. Microarray data are mean values over all time points: 24 h, 48 h, 72 h, 96 h) of sulfur starvation. Probes marked with a star are not specific for the transcript model.

To analyze the specificity of the array system we used the knock out mutant *npq*4 (kind gift of Prof. Krishna Niyogi, University of California), which has been previously shown to be deficient in the *LHCSR3 *gene transcripts [38], to check if we can detect the genotype on the RNA level. It is known that the *LHSCR1*, *LHCSR3.1 *and *LHSCR3.2 *gene expression is enhanced under sulfur starvation and/or during hydrogen production induced by sulfur deprivation [23]. Therefore, we analyzed the transcript level for *npq*4 and the parental strain *4A+ *under sulfur starvation. The expression rates under sulfate starvation increased for all three transcripts in the wild type, but no gene expression was detectable in the *npq4 *mutant in the *LHCSR3.1 and LHSCR3.2 *isoforms, as expected (see Figure 5). For this reason, we can state a high specificity for our designed probes regarding the *LHCSR *transcripts and in combination with the analyzed nutrient starvation experiments a good usability of our microarrays. Comparison with WT *cc3491 *grown under sulfur deprivation, showed no significant changes regarding the normal response to sulfur starvation.

## Discussion

In this work we successfully designed and tested a novel microarray platform for *Chlamydomonas reinhardtii*. We were able to determine unique sequences for most (93%) of the transcript models obtained from the *Joint Genome Institute *data base version JGI 3.1. For a small number of transcript models (7%) we could not identify a specific gene sequence and consequently these genes cannot be analyzed with the current microarray. We confirmed the specificity for 11000 probe sets with the current annotation (AUGUSTUS 10.2). We tested our microarray with RNA samples from cultures grown under different nutrient stress conditions and compared our data with recent publications. By doing this, we could confirm with our new system previously published changes in gene expression during nutrient starvation for many genes and hereby proved that this newly designed array is very useful for general transcription analysis. In addition, RNA amplification enabled us to detect several low abundant regulator genes expressed under nutrient starvation with the acquired data being in good accordance with previously published RNA-seq and microarray data [16,17]. Microarrays are cheap and reliable tools for monitoring transcript changes, and although RNA-seq methods may allow a more detailed view inside the transcriptome, the lower costs, the high reproducibility and the established analysis systems advantages microarrays for routine applications. Therefore, one can now choose the platform, which provides the best conditions for the individual experiment.

In functional tests of the microarrays we were able to show, besides proof of functionality for the analysis of differential gene regulation under nutrient stress conditions, the deletion of the *LHCSR3.1 *and *LHCSR3.2 *in the knockout strain *npq4 *[38] . This result let us conclude that the platform is sensitive to investigate knock out or knock down strains. The successful confirmation of the lack of the corresponding transcript and of induction of expression under sulfur stress for *LHCSR3.1/LHCSR3.2 *clearly proved the suitability of the array for the analysis of gene deletions. Furthermore, as the probes did not show any cross hybridization or increased unspecific binding, the specificity of the designed probes has clearly been demonstrated in this work. Intriguingly, the *LHCSR1 *genes did not show an up-regulation during nitrogen starvation in our work, thus confirming previous experiments [17].

Under nutrient starvation the first response of the cells is an up-regulation of transport systems required for the specific nutrient. We were able to prove gene expression of such transporters for both, nitrogen and sulfate starvation as well as the increased expression for other nitrogen and sulfate specific genes. Both starvation conditions resulted in an increase of transcript levels already after 12 hours of nutrient depletion. These higher levels of transcription rates were consistent throughout the whole time of the experiment. Differences between our and previous studies [16] could be a result of the longer starvation period and use of different *Chlamydomonas *strains *cc125 *and *cc3491 *instead of *D66*, *ars11, 21gr*. Additionally we included into our analysis just those genes with an up-regulation monitored within all time points. Therefore, differences in gene expression level and the number of genes vary.

## Conclusion

In summary, our data proved a high sensitivity of the array as a precondition for further detailed and advanced transcript analyses of mutant *vs *wt strains in *C. reinhardtii*. With probes for 14557 transcript models (11000 in AUGUSTUS 10.2) our new array offers a very good coverage of the *Chlamydomonas *genome. It is noteworthy that current genome annotation predictions estimate around 12000-17000 gene models, so most likely some transcript models are still missing on the platform, however an updated version of the array can easily be established in the future.

## Methods

### Genome annotation and Probe design

DNA sequences for around 15000 predicted transcript models were obtained from the *Joint Genome Institute *(JGI 3.1 and AUGUSTUS 5.0 http://genome.jgi-psf.org/chlamy/chlamy.home.html) and several new proteome findings were included. Oligonucleotides that represent the transcriptome of *C. reinhardtii *were designed using Agilent array probe design software (ARRAY EXPRESS^©^, see https://earray.chem.agilent.com/earray/) and an open source program (PROMIDE^©^, CeBiTec, Bielefeld). A third probe per transcript was added, which was designed based on the first microarray platform [2]. In summary we used three independent probes per transcript. The expected melting temperature and the G+C content for all probes were determined as described in [39]. The microarrays were produced by *Agilent^© ^*and are available under the array number 024664. The determined probe sequences were mapped to the newer transcript models using BLAST alignments [40]. A probe with a sequence aligning perfectly to only one transcript sequence, not aligning to any other transcript sequence while allowing for up to 3 mismatches, was considered specific to that transcript model.

The *Chlamydomonas *full genome microarray (*Agilent^© ^*no.: 024664) matrix, sequences, row data and normalized data are deposited in the GEO database http://www.ncbi.nlm.nih.gov/projects/geo/ with the accession number (GSE33042).

### RNA preparation

Samples taken from bioreactors (300 mL) were immediately centrifuged 83000 *g*, 2 minutes at room temperature). Fresh cell pellets were lyzed immediately with RNA Lysis Buffer and RNA was isolated as previously described [20].

### Microarray preparation and data acquisition

*C. reinhardtii *microarray slides (Agilent^© ^4 × 44 k, no: 024664) were used for the transcript analysis. RNA labeling (Quick RNA amplification and labeling kit; *Agilent*) and microarray hybridization (16 h at 60°C) were carried out according to the supplied manual.

### Microarray scanning and data analysis

The microarrays were washed after hybridization according to the Agilent^© ^manual, dried in a centrifuge and scanned with a 5 μm resolution in a high resolution Agilent^© ^DNA microarray scanner. Data extraction was achieved using the feature extraction software (10.7.3.1; Agilent^©^) and data were normalized and analyzed using EMMA2, an open source software application for microarray data analysis [32,41]. We used a robust normalization method (lowess) and we performed significance tests within all experiments and considered only those probes showing a significant change in their expression (p-values smaller than 0.05). To account for the multiple testing situations, all computed p-values were corrected using the method of Holm-Bonferroni [32,41]. To further limit our result set, we included in our analysis only those genes that showed at least a two-fold up- or down-regulation.

### Strains

The following *C. reinhardtii *strains were used: wild type *cc125 *and *cc3491*. The non-photochemical quenching mutant npq4 was generated by insertional mutagenesis [38], resulting in a knockout of the *LHCSR3.1 *and *LHCSR3.2 *genes [33]. The *4A+ *wild-type strain [42] was used as the control in experiments involving npq4.

Cultures were grown in normal TAP media till early stationary phase and re-suspended after washing (3 times) in TAP minus S or N media and cultivated for 96 h under constant light (200 μE m^-2 ^s^-1^).

### Samples collections

Samples from *C. reinhardtii cc3491 *from five time points were collected at 0 h, 24 h, 48 h, 72 h and 96 h after sulfur/nitrogen starvation (T0, T1, T2, T3 and T4 respectively). Reference samples (T0) were harvested from early stationary phase cultures of the corresponding strain before starvation. *npq4 *and *4A+ *were cultivated under sulfate starvation conditions and samples were taken every 24 h for 96 h.

## Authors' contributions

JT carried out the microarray design, the sulfur stress experiments and drafted the manuscript. SPA and AG performed the statistical analysis, SA carried out the sequence alignment. MIR and KR participated in the sulfur and nitrogen stress experiments. OK conceived of the study, and participated in its design and coordination and helped to draft the manuscript. All authors read and approved the final manuscript."

## Supplementary Material

Additional file 1 **Table S1:Transcript specific sequences for the new *Chlamydomonas reinhardtii *full genome microarray**. Probe identifier, probes sequence and origin for the *Chlamydomonas reinhardttii *4*44 K Agilent microarray. Click here for file

Additional file 2 **Table S2. Confirmation of the Sequence specificity compared to the newest annotation (Augustus 10.2)**. Specificity for 60 mer oligonucleotides designed for the Chlamydomonas reinhardttii 4*44 K Agilent microarray with three replicates per transcript model in comparison to AUGUSTUS 10.2 annotation (Description: single: Probe is specific for one specific transcript in the used annotation version, multiple: Probe is not specific to one transcript, but to multiple transcripts in the used annotation version. None: probe is not specific to any transcripts in the used transcriptome version.). A summary for all transcript models with the corresponding specific probes (up to three independent probes per transcript) is given in a second Table. Click here for file

Additional file 3 **Table S3. Gene expression of *Chlamydomonas reinhardtii *under nitrogen starvation**. Comparison between RNA-seq [17] (48 h) and microarray data for *Chlamydomonas reinhardtii *cultures incubated under nitrogen starvation conditions (24 h, 48 h, 72 h, 96 h). Click here for file

Additional file 4 **Table S4. Gene expression of *Chlamydomonas reinhardtii *under sulfur starvation**. Comparison between RNA-seq [16] (48 h) and microarray data for *Chlamydomonas reinhardtii *cultures incubated under sulfur starvation conditions (24 h, 48 h, 72 h, 96 h). Click here for file

Additional file 5 **Table S5. Gene expression of *Chlamydomonas reinhardtii *under nutrient starvation**. Transcript levels for genes with differential expression in *Chlamydomonas reinhardtii *cultures incubated under sulfur and nitrogen starvation conditions (24 h, 48 h, 72 h, 96 h). Click here for file
